# Thyroid Function in Chronically Transfused Children with Beta Thalassemia Major: A Cross-Sectional Hospital Based Study

**DOI:** 10.1155/2018/9071213

**Published:** 2018-09-16

**Authors:** Suraj Haridas Upadya, M. S. Rukmini, Sowmya Sundararajan, B. Shantharam Baliga, Nutan Kamath

**Affiliations:** ^1^Mission Hospital, Mysuru, India; ^2^Department of Biochemistry, Kasturba Medical College, Mangalore, Manipal Academy of Higher Education, India; ^3^Kasturba Medical College, Mangalore, Manipal Academy of Higher Education, India; ^4^Department of Paediatrics, Kasturba Medical College, Mangalore, Manipal Academy of Higher Education, India

## Abstract

**Background:**

Thalassemia is the most common genetic disorder worldwide. Use of iron chelators has improved survival but endocrine complications have become more frequent. The frequency of hypothyroidism in Beta Thalassemia Major (BTM) children ranges from 6 to 30 %. Thyroid dysfunction mainly occurs by gland infiltration, chronic tissue hypoxia, free radical injury, and organ siderosis.

**Objectives:**

(a) To evaluate the thyroid function status in chronically transfused children with BTM, in the first and second decade of life and (b) to study the influence of factors like duration and amount of blood transfusions, serum ferritin level, and iron chelation therapy on thyroid function.

**Methodology:**

BTM children, 3 years old and above, on regular blood transfusions with serum ferritin > 1500 mcg/l were included in the study. Thyroid function and ferritin assessment was done using ELISA kits. Autoimmune thyroiditis was ruled out by antithyroid peroxidase and antithyroglobulin antibody testing.

**Results:**

A study population of 83 children consisted of 49 boys (59%) and 34 girls (41%). 4.8% of the children had evidence of subclinical hypothyroidism. Among them two belonged to the first decade and the other two to the second decade of life. Mean TSH, FT4, and ferritin values among children with thyroid dysfunction were 6.38 ± 0.83 mIU/ml, 1.08 ± 0.45 ng/dl, and 3983.0±1698.30 ng/ml, respectively. The severity of thyroid dysfunction was statistically significantly associated with higher serum TSH values in children in the second decade of life with a p value = 0.001. No other significant correlation was found between oral chelation, amount and duration of blood transfusion, or serum ferritin levels.

**Conclusion:**

Subclinical hypothyroidism was the thyroid dysfunction observed in our study. Regular blood transfusions with adequate chelation may decrease incidence of thyroid dysfunction.

## 1. Introduction

The most common genetic disorder in the world is known to be thalassemia [1]. Beta thalassemia syndromes are disorders which are inherited and are characterized by deficiency in the production of beta globin chains resulting in ineffective erythropoiesis complicated by lack of affinity of circulating haemoglobin F to 2,3-diphosphoglycerate. As a consequence of this, repeated blood transfusions are needed to maintain life, which in turn results in excessive iron being deposited in various organs resulting in early fatalities. In Beta Thalassemia Major (BTM) patients the frequency of hypothyroidism ranges from 6 to 30 % among various countries depending on chelation strategies [2]. The quantity and duration of iron overload mainly determine the prognosis among such patients. Thyroid dysfunction mainly occurs by gland infiltration, chronic tissue hypoxia, free radical injury, and organ siderosis. The thyroid gland is affected much before the thyroid-pituitary axis, which is less susceptible than the gonadal axis to iron induced damage [3]. In severe hemosiderosis, the anterior pituitary may be affected resulting in disruption of regulatory hormonal secretions (LH, FSH, and TRH) [4]. With the introduction of iron chelators, survival rates have improved [5] but endocrine complications have become more frequent and significantly affect the quality of life [6]. The symptoms of hypothyroidism though non-specific can affect many organ systems, hence an annual laboratory evaluation of thyroid function is recommended in all beta thalassemics. Thyroid functions in chronically transfused children with BTM in the second decade of life have been studied, but there are very few studies evaluating the thyroid function in chronically transfused children in the first decade of life. Therefore, a comparative study to evaluate the thyroid function status in children with BTM in the first and second decade of life is the need of the hour.

## 2. Methodology

A hospital based cross-sectional study was conducted at Kasturba Medical College (KMC) Hospital, Attavar & Regional Advanced Paediatric Care Centre (RAPCC), Government Wenlock Hospital, Mangalore. 83 diagnosed cases of BTM children aged 3 years and above who were on regular blood transfusions were included in the study. The study was conducted for a 2-year period from October 2013 to September 2015. A sample size of 83 was calculated considering the prevalence of hypothyroidism in chronically transfused children with BTM to be 17.6% [7] with 95% confidence limits and 80% power. Applying the formula for finite population, n = n0/ (1 + n0/N) where N=83 as according to available hospital records, the sample size was calculated to be 83. All diagnosed cases of BTM children aged 3 years and above who were on regular blood transfusions (10-15 ml/kg every 2-4 weeks) for at least 1 year with a serum ferritin value >1500 mcg/l [8] were included in the study. Children with beta thalassemia intermedia, beta thalassemia minor, BTM children receiving thyroid/antithyroid supplements, family history of hypothyroidism, and those with other acute illnesses were excluded from the study. Patients who fulfilled the inclusion criteria were recruited into the study after getting clearance from the institutional ethics committee. Informed consent was obtained from either of the parents of the child and a semistructured proforma was prepared to record all data. Patients were instructed to avoid any other medications (apart from chelators) prior to transfusion. 3ml of random venous blood samples was collected in a plain vacutainer under sterile aseptic precautions from all the patients on the day of admission prior to the start of blood transfusion for the estimation of serum free thyroxine, thyroid stimulating hormone, and serum ferritin levels. Samples were stored in the deep freezer at -20°C. Samples were processed on a single day in the Biochemistry Department of Centre for Basic Sciences KMC Bejai using Weldon Biotech ELISA kit for the estimation of FT4, TSH, and ferritin. Antithyroglobulin and antithyroid peroxidase antibody titers were estimated in patients with TSH >5 mIU/L [9] by Electrochemiluminescence Immunoassay (ECLIA). Thyroid dysfunction was defined as follows: Overt Primary Hypothyroidism (FT4< 0.7 ng/dL with TSH > 5 mIU/L), Compensated Hypothyroidism (Normal FT4 with TSH > 5mIU/L), and Secondary Hypothyroidism (FT4 < 0.7 ng/dl with low or normal TSH) [7]. Data entry and analysis were done using SPSS (Statistical Package for Social Sciences) Version 17. Basic sociodemographic data of the study participants were expressed in terms of frequency and percentage for categorical variables and means with standard deviation for continuous variables. Serum levels of Vitamin D were expressed in means with standard deviations. Association between thyroid dysfunction and important variables (age, gender, weight, height, oral chelation, frequency of transfusions, and serum ferritin) were tested using Chi square test and Fischer's Exact test, and paired t test. p value less than 0.05 was considered as significant and less than 0.01 as highly significant.

## 3. Results

A study population of 83 children consisted of 49 boys (59%) and 34 girls (41%). Among them 44 (43%) belonged to the first decade and 39 (47%) to the second decade of life (Table 1). Anthropometric assessment was conducted for all children included in this study and found that 63.9 % were underweight and 66.3 % stunted for age (Table 1). 4.8% of the children had evidence of subclinical hypothyroidism. Among them two belonged to the first decade and the other two to the second decade of life. None of the BTM children with thyroid dysfunction had autoimmune thyroiditis. Mean TSH, FT4, and ferritin values among children with thyroid dysfunction were 6.38 ± 0.83 mIU/ml, 1.08 ± 0.45 ng/dl, and 3983.0±1698.30 ng/ml, respectively (Table 2). The severity of thyroid dysfunction was statistically significantly associated with higher serum TSH values in children in the second decade of life with a p value = 0.001. No other significant correlation was found between oral chelation, amount and duration of blood transfusion, or serum ferritin levels.

## 4. Discussion

BTM children on regular transfusions and suboptimal chelation are at an increased risk for iron overload. Like in all organs, iron is deposited in the thyroid interstitium resulting in thyroid hemosiderosis. This slowly leads to worsening of the thyroid function. This study aims to analyze the type of thyroid dysfunction and its association with duration and amount of blood transfusions, serum ferritin level, and adequacy of chelation. Iron deposition from repeated transfusions has been implicated as the likely mechanism causing thyroid dysfunction in BTM patients. Serum ferritin has a direct correlation to iron accumulation in the liver. Serum ferritin being an acute phase protein is also a product of hepatocellular damage. As a result, sepsis, congestive heart failure, and hepatitis can result in a falsely elevated measurement. No patients involved in our study have any clinical evidence of hepatitis or heart failure.

Our study aimed at evaluating thyroid function in chronically transfused children with Beta Thalassemia Major in the first and second decade of life. The study population consisted of 49 boys (59%) and 34 girls (41%). The influence of factors like duration and amount of blood transfusions, serum ferritin level, and iron chelation therapy on thyroid function was evaluated. Thyroid function was assessed using ELISA kits for the estimation of TSH, Free T4, and the iron overload was estimated by measuring the serum ferritin levels. In those patients with evidence of thyroid dysfunction, autoimmune thyroiditis was ruled out by estimating levels of antithyroglobulin and antithyroperoxidase by ECLIA method. In this study 44 children (43%) belonged to the first decade and 39 children (47%) belonged to the second decade of life (Table 1). Among them 41% were females and 59% were males (Table 1). Anthropometric assessment was also conducted for all children included in this study and found that 63.9 % were underweight and 66.3 % stunted for age (Table 1). 78.3% children were on regular transfusions (Table 1). 56.6% of children were diagnosed to have thalassemia before the age of 1 year (Table 1). 44% of children were on regular oral chelation for a period of less than 5 years (Table 1). Subclinical hypothyroidism was the most common thyroid dysfunction observed in our study.

No correlation was noticed between thyroid dysfunction and age in contrast to studies done by Garadah [10], Mula Abed WA [11], Filosa A [9], Karamifar H [12], and Zervas [13] where thyroid dysfunction was seen in the second decade of life (Table 1). A significant correlation was noticed between thyroid dysfunction and weight for age (Table 1), whereas height did not yield any significant association (Table 1) which is in contrast to a study done by Peiman Eshragi et al. [14] where they found statistical significance between height and thyroid dysfunction. No correlation was found between gender, oral chelation, amount and duration of blood transfusion, and age at diagnosis with pattern of thyroid dysfunction (Table 1). In our study, 4 out of 83 children (4.8%) had evidence of subclinical hypothyroidism with elevated levels of TSH. Among them one was a female and the other two were males. Two children belonged to the first decade and the other two to the second decade of life. All the 4 children received regular blood transfusions and oral iron chelation therapy. This is similar to other published studies done by Sharma et al. [15] and Abdel-Razek et al. [16] where subclinical hypothyroidism was most frequently reported. There was no evidence of autoimmune thyroiditis in children with thyroid dysfunction in our study. Few studies like the ones done by Soliman AT et al. [17] and De Sanctis et al. [18] showed prevalence of Central Hypothyroidism among BTM children in the second decade of life.

All the children with thyroid dysfunction had high serum ferritin levels despite chelation with a mean ferritin value of 3983.0±1698.30 ng/ml (Table 2), similar to a study done by Aydinok [19] where a mean ferritin value of 3597 ± 1931 ng/ml was observed in the hypothyroid group. This is in contrast to studies done by Zervas [13] and Ayfer Gozu Pirinccioglu [20] where the mean ferritin values among the hypothyroid group were 2707.66 ± 1990.5 ng/ml and 2703 ± 1649 ng/dl, respectively. But there was no statistical significance noticed between serum ferritin levels and pattern of thyroid impairment (p value of 0.806) consistent with the studies done by Soliman et al. [17] and Zervas [13] and in contrast with the study done by Chirico et al. [21] which proved ferritin as a prognostic marker for BTM patients and a predictive factor for progression to thyroid dysfunction. The mean TSH value among the thyroid dysfunction group was 6.38 ± 0.83 mIU/ml which is similar to a study done by Garadah [10] on thalassemic patients where the mean TSH value was 6.78 ± 1.5mIU/ml; however studies done by Abdel Razek [16] and Somchit Jaruratanasiriku [7] showed mean TSH values of 4.5±4.8 mIU/ml and 5.9±15.6 mIU/ml, respectively, among children with subclinical hypothyroidism. Statistical significance was found between TSH and thyroid dysfunction (p value= 0.001) (Table 2), consistent with the study done by Garadah [10] with a p value < 0.01. The mean Free T4 value among the thyroid dysfunction group was 1.08 ± 0.45 ng/dl.

Since our study has shown an association between deteriorating thyroid function and iron overload, improving the compliance to chelation, changing the chelator used was necessary and regular monitoring of thyroid function from the first decade of life will have a significant change in arresting the thyroid hemosiderosis and the thyroid dysfunction thereafter. Further, annual evaluation of thyroid function in BTM children and the initiation of hormone replacement if found deficient will be beneficial.

## 5. Conclusion

The most common abnormality in thyroid function in chronically transfused BTM children is subclinical hypothyroidism. No correlation was found between thyroid dysfunction and factors like duration and amount of blood transfusions, serum ferritin level, and iron chelation therapy. Subclinical hypothyroidism was found in 43 % of the study cohort in the first decade of life though not statistically significant. Thyroid dysfunction may be seen in adolescents with subclinical hypothyroidism with frequent blood transfusions.

## Figures and Tables

**Table 1:** 

	**Frequency**	**Percent**	**Thyroid Dysfunction (Present)**
**Age distribution**			

Below 5 years	8	9.6	0
5 years–9 years	36	43.4	2
Above 10 years	39	47	2

**Height distribution**			

< 3RD	55	66.3	2
10TH-25TH	6	7.2	0
10TH-3RD	5	6.0	0
10TH	2	2.4	0
25TH-50TH	1	1.2	0
25TH	4	4.8	1
3RD-10TH	5	6.0	0
3RD	2	2.4	0
50TH-25TH	1	1.2	0
50TH	2	2.4	1

**Total**	**83**	**100.0**	**4**

**Weight distribution**			

<3RD	53	63.9	1
10TH-25TH	4	4.8	0
10TH-3RD	5	6.0	0
10TH	4	4.8	0
25TH-50TH	3	3.6	1
25TH	3	3.6	0
3RD-10TH	1	1.2	0
3RD	6	7.2	1
50TH-25TH	1	1.2	1
50TH	3	3.6	0

**Total**	**83**	**100.0**	**4**

**Gender distribution**			

Female	34	41	1
Male	49	59	3

**Transfusion**			

Monthly	65	78.3	4
Bi-monthly	2	2.4	0
Tri-monthly	15	18.1	0
Once in 40 days	1	1.2	0

**Age at diagnosis**			

>1 year	47	56.6	2
1 year–5 years	31	37.3	2
Above 5 years	5	6.0	0

**Oral Chelation Duration**			

Below 5yrs	44	53.0	4
5yrs and above	34	41.0	0
NA	5	6.0	0

**Total**	**83**	**100.0**	**4**

**Table 2:** 

**Thyroid Dysfunction**	**Frequency**	**Mean**	**Standard deviation**	**Mann-Whitney U Test**
**Evaluation of thyroid dysfunction by ferritin**				

Present	4	3983.00	1698.30	.805
Absent	79	4322.13	2700.63	NS

**Evaluation of thyroid dysfunction by TSH**				

Present	4	6.38	.83	.0001
Absent	79	2.68	.91	HS

**Analysis of thyroid function with TSH, Free T4 and ferritin values in the second decade of life**				

**Dosage (mg/kg/day)**				

Present	2	23.00	2.83	0.948
Absent	36	23.33	7.09	

**Ferritin (ng/ml)**				

Present	2	4947.50	1283.40	0.959
Absent	38	4846.11	2723.12	

**TSH (mIU/ml)**				

Present	2	6.85	.52	.0001
Absent	38	2.51	.90	

**Free T4 (ng/dl)**				

Present	2	1.23	.73	0.141
Absent	38	1.52	.25	

## Data Availability

The data used to support the findings of this study are included within the article.
